# First case report of rheumatoid-like paraneoplastic polyarthritis in a patient with Fallopian tube cancer

**DOI:** 10.1093/rap/rkad041

**Published:** 2023-04-19

**Authors:** Guido Lewik, Lena S Müller, Gerrit Lewik

**Affiliations:** Translational Research Immunology Group, Nuffield Department of Surgical Sciences, University of Oxford, Oxford, UK; Department of Orthopedic and Trauma Surgery, Katholisches Klinikum Bochum—St. Josef Hospital, Ruhr University Bochum, Bochum, Germany; Department of General and Trauma Surgery, BG University Hospital Bergmannsheil, Ruhr University Bochum, Bochum, Germany; Department of General and Trauma Surgery, BG University Hospital Bergmannsheil, Ruhr University Bochum, Bochum, Germany

Key message

- Atypical arthritis warrants consideration of underlying malignancies and advising regular gynaecological check-ups, especially before anti-TNF therapy.

Dear Editor, Paraneoplastic (poly-)arthritis (PA) is a rarely reported side effect of the antitumour immune response, preceding cancer manifestation by a mean of 10.7 months [1]. Diagnosed *ex juvantibus*, if at all, and with <150 cases reported worldwide showing a marked heterogeneity in clinical presentation and underlying tumour entities, timely recognition is extremely complicated. Nevertheless, signs such as rapid onset and resistance to therapy should raise suspicion, and correct identification could expedite cancer diagnosis and treatment. Here, we present the case of a woman who, after 20 months of treatment for RA with little effect, experienced full symptom resolution upon diagnosis and removal of a Fallopian tube carcinoma.

A 63-year-old, non-smoking, lean Caucasian woman with an active lifestyle presented for a rheumatology consultation with new onset of morning stiffness and painful joint swelling, predominately affecting her hands, which hindered her from pursuing leisure sports and everyday activities. Past medical history and medication merely revealed valsartan-treated arterial hypertension. Physical examination showed swollen, warm and painful MCP and basal thumb joints of both hands. Her further clinical impression was unremarkable. Blood tests returned positive results for RF (29.6 U/ml), CCP (46.0 U/ml) and ANA (1:320), while the inflammatory markers ESR and CRP were normal.

Initially fulfilling diagnostic criteria for RA and with symptoms refractory to early treatment with etoricoxib, she was started on MTX and received complementary prednisolone. Only marginal reduction in symptoms led to MTX up-titration over time, until elevated liver enzymes prompted a switch to LEF at ∼1 year of treatment (Fig. 1). Repeated X-ray imaging showed no signs of joint destruction, and DXA indicated normal bone density. The markers of inflammation ESR and CRP also remained at normal levels despite high arthritis disease activity. After several more months of therapy with LEF and intermittent prednisolone, which yielded unsatisfactory results, her treatment was escalated to TNF blockade with etanercept (Fig. 1). After a few weeks with remaining, albeit alleviated, complaints of arthritis using this regimen, a delayed routine gynaecological US examination revealed an irregular mass in her lower abdomen.

**Figure 1.**
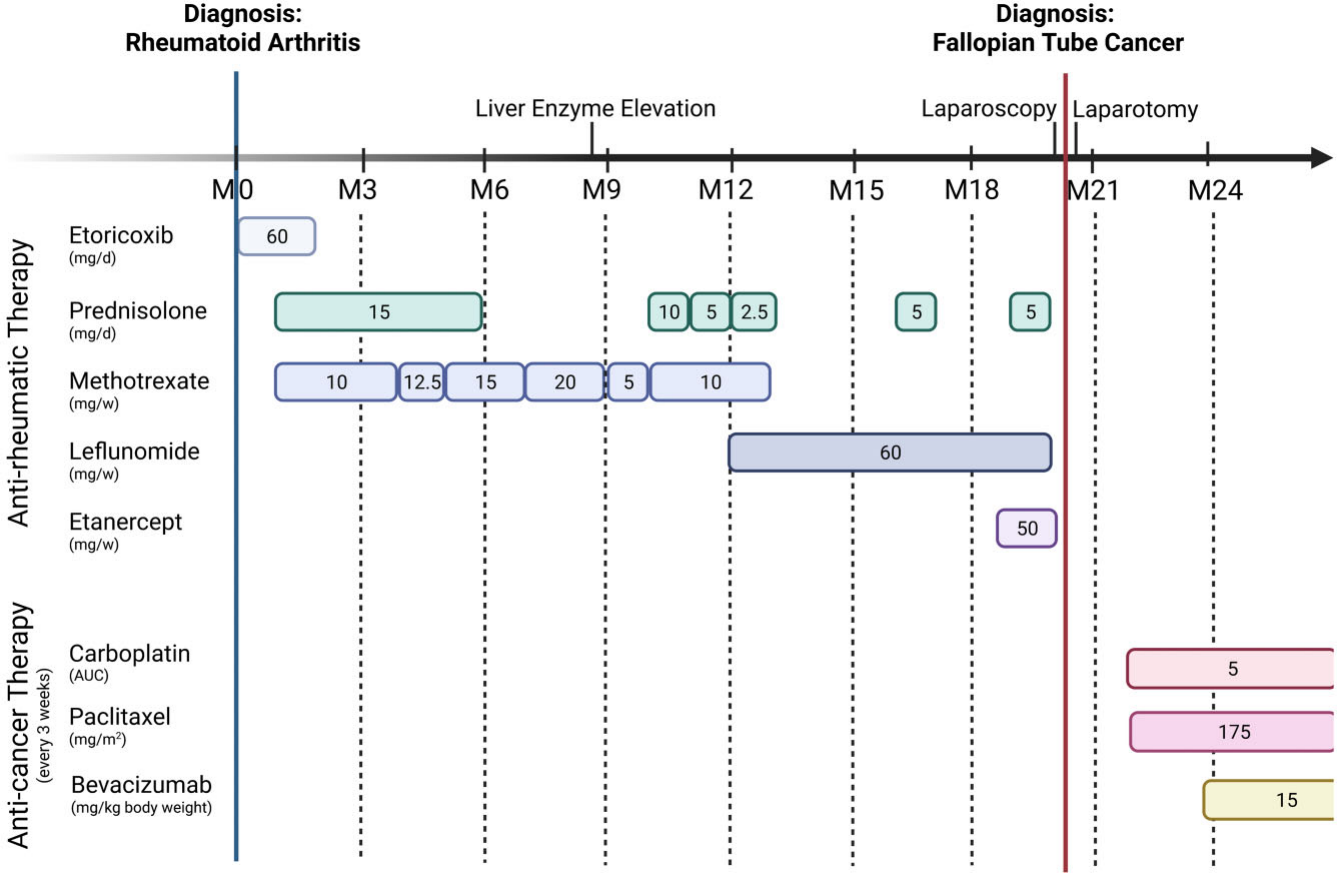
Timeline of main events and therapy regimens against arthritis and cancer. Created with BioRender.com

Immunosuppressive medication was stopped owing to suspected malignancy, and timely pelvic MRI substantiated the impression of a tumour. The patient was admitted to a specialized gynaecological cancer centre and underwent laparoscopy, with bilateral adnexectomy and partial removal of omentum majus and peritoneum. Pathohistological analysis revealed a high-grade serous papillary adenocarcinoma originating from the right Fallopian tube, with disseminated omental and peritoneal metastases. Notably, symptoms of arthritis vanished completely upon laparoscopic tumour debulking. Subsequent laparotomy achieved optimal cytoreductive surgery with no residual disease, resulting in International Federation of Gynecology and Obstetrics (FIGO) stage IIIc (TNM classification: pT3c pN1b(2/4) L1 V0 Pn0 M0).

After postoperative recovery, the patient was started on adjuvant chemotherapy with six cycles of carboplatin and paclitaxel every 3 weeks, and addition of bevacizumab every 3 weeks for 15 months as per interdisciplinary tumour board recommendation (Fig. 1). She has now been off immunosuppressive medication for almost 6 months, and no symptoms of arthritis have re-emerged despite a return to prior daily activities and moderate exercise.

Many features of this case are in line with prior PA literature, namely occurrence as symmetrical polyarthritis in a patient >50 years of age showing poor response to standard therapy with NSAIDs, glucocorticoids and DMARDs, yet complete vanishing of joint complaints after cancer treatment [2–4]. However, PA patients are usually male and seronegative for RF and CCP, which are detectable in only 23% and 11%, respectively [2, 5].

Importantly, PA relief upon tumour removal in this patient was sustained without medication over 6 weeks between debulking and chemotherapy commencement, thereby far exceeding any prior arthritis-free interval and prompting reclassification from RA to (rheumatoid-like) PA. Continued absence to date confirms its paraneoplastic aetiology, disputing symptom fluctuations or intermittently reduced physical activity as confounders.

Notably, PA has most often been found in lung adenocarcinoma and haematological malignancies [2], and the literature yields only two prior reports of adnexal tumour association: one ovarian teratoma in a 34-year-old woman [6] and one ovarian cystadenocarcinoma in a 64-year-old woman, who, however, had an additional malignant sarcomatoid lung tumour [7].

This case report thus describes, for the first time, rheumatoid-like PA in a patient with Fallopian tube cancer. Towards improved patient care, this addition to the sparse data on PA is relevant for two main reasons. Firstly, increased awareness of PA could address the anticipated problem of underreporting, considering its solely retrospective diagnosis, the fact that advanced cancer will not always respond to treatment well enough for PA reversion and that concomitant circumstances might overshadow the follow-up of co-morbidities. Indeed, a multicentre study from France indicated that, although PA is considered rare, it made up 2.6% of admitted early arthritis patients [8]. Secondly, awareness of PA could aid patients with atypical rheumatological presentation by a mere emphatic reminder to adhere to regular routine checks, such as gynaecological examinations, offering a low-/no-cost opportunity to find and treat malignancies sooner.

## Data Availability

The data underlying this article will be shared on reasonable request to the corresponding author.
